# Two sirtuin proteins, Hst3 and Hst4, modulate asexual development, stress tolerance, and virulence by affecting global gene expression in *Beauveria bassiana*

**DOI:** 10.1128/spectrum.03137-23

**Published:** 2024-01-09

**Authors:** Qing Cai, Li Tian, Jia-Tao Xie, Dao-Hong Jiang

**Affiliations:** 1 College of Plant Science and Technology, State Key Laboratory of Agricultural Microbiology, Huazhong Agricultural University, Wuhan, Hubei, China; 2 Department of Bioengineering, Shandong Provincial Key Laboratory of Microbial Engineering, Qilu University of Technology, Jinan, Shandong, China; Agroscope, Nyon, Switzerland

**Keywords:** sirtuins, asexual development, stress tolerance, DNA damage response, virulence, gene transcription

## Abstract

*Beauveria bassiana* is a widely used entomopathogenic fungus in insect biological control applications. In this study, we investigated the role of two sirtuin homologs, BbHst3 and BbHst4, in the biological activities and pathogenicity of *B. bassiana*. Our results showed that deletion of *BbHst3* and/or *BbHst4* led to impaired sporulation, reduced (~50%) conidial production, and decreased tolerance to various stresses, including osmotic, oxidative, and cell wall-disturbing agents. Moreover, BbHst4 plays dominant roles in histone H3-K56 acetylation and DNA damage response, while BbHst3 is more responsible for maintaining cell wall integrity. Transcriptomic analyses revealed significant changes (>1,500 differentially expressed genes) in gene expression patterns in the mutant strains, particularly in genes related to secondary metabolism, detoxification, and transporters. Furthermore, the Δ*BbHst3*, Δ*BbHst4*, and Δ*BbHst3*Δ*BbHst4* strains exhibited reduced virulence in insect bioassays, with decreased (~20%) abilities to kill insect hosts through topical application and intra-hemocoel injection. These findings highlight the crucial role of BbHst3 and BbHst4 in sporulation, DNA damage repair, cell wall integrity, and fungal infection in *B. bassiana*. Our study provides new insights into the regulatory mechanisms underlying the biological activities and pathogenicity of *B. bassiana* and emphasizes the potential of targeting sirtuins for improving the efficacy of fungal biocontrol agents.

**IMPORTANCE:**

Sirtuins, as a class of histone deacetylases, have been shown to play important roles in various cellular processes in fungi, including asexual development, stress response, and pathogenicity. By investigating the functions of BbHst3 and BbHst4, we have uncovered their critical contributions to important phenotypes in *Beauveria bassiana*. Deletion of these sirtuin homologs led to reduced conidial yield, increased sensitivity to osmotic and oxidative stresses, impaired DNA damage repair processes, and decreased fungal virulence. Transcriptomic analyses showed differential expression of numerous genes involved in secondary metabolism, detoxification, transporters, and virulence-related factors, potentially uncovering new targets for manipulation and optimization of fungal biocontrol agents. Our study also emphasizes the significance of sirtuins as key regulators in fungal biology and highlights their potential as promising targets for the development of novel antifungal strategies.

## INTRODUCTION

Histone acetylation is a post-translational modification of histone proteins that plays a crucial role in regulating gene expression and various chromatin-mediated DNA processes (1). Histone acetylation is primarily catalyzed by enzymes known as histone acetyltransferases (HATs) and histone deacetylases (HDACs) (2, 3). HATs can add acetyl groups to specific lysine residues on histone tails, leading to a more open chromatin structure that facilitates gene transcription (2). On the contrary, HDACs remove acetyl groups, resulting in a more compact chromatin conformation associated with gene repression (3). HDACs are categorized into four classes based on their sequence homology. The two major classes are class I and class II HDACs, which include enzymes like Rpd3/Hda1-like HDACs (4). Additionally, there is a class III HDAC family known as sirtuins, which require nicotinamide adenine nucleotide (NAD^+^) as a co-factor for their deacetylase activity (5). These NAD^+^-dependent sirtuins are involved in diverse cellular processes such as gene expression regulation, maintenance of heterochromatin, genome stability, and control of replicative lifespan (6–8).Hst3 and Hst4 are two members of the sirtuin family of NAD^+^-dependent HDACs, which are found in virtually all organisms (9). Hst3 and Hst4 are responsible for deacetylating lysine 56 (K56) on histone H3 (10, 11). Proper acetylation of H3-K56 is essential for cell survival, DNA replication, and nucleosome assembly (12, 13). In *Saccharomyces cerevisiae*, deletion of *Hst3* and *Hst4* leads to constitutive acetylation of H3-K56 and results in defects in cell cycle progression, chromosome loss, DNA damage, and increased sensitivity to genotoxic agents (10, 14). In *Schizosaccharomyces pombe*, knockdown of Hst4 is accompanied by increased H3-K56 acetylation during the cell cycle, DNA damage, and oxidative stress (15, 16). These findings suggest that Hst3 and Hst4 play a crucial role in maintaining genome integrity and in regulating H3-K56 acetylation in response to cellular stresses.

Sirtuin families in filamentous fungi are homologous to Sir2, Hst1, Hst2, Hst3, and Hst4 in *S. cerevisiae*; these fungal sirtuins have diverse roles in fungal development, secondary metabolism, stress response, and virulence (17). In *Candida albicans*, *Hst3* is an essential gene, and its conditional mutants show attenuated virulence in mice (18). Also, the knockdown of *Hst3* leads to alterations in the switching between white and opaque cell phenotypes, which are associated with the virulence of this fungus (19). In *Cryptococcus neoformans*, both Hst3 and Hst4 are required for full virulence in a murine infection model; the loss of *Hst3* and *Hst4* results in reduced pathogenicity (20). In *Aspergillus nidulans*, the SirE protein (homologous to Hst3) regulates primary metabolic processes, cell wall synthesis, and the transition from primary growth to the stationary phase (21). In *Aspergillus oryzae*, Hst4/HstD is important for stress response, drug resistance, secondary metabolism, and development (22, 23). In *Magnaporthe oryzae*, the deletion of *MoHst4* leads to reduced conidiation (asexual spore production) and impaired infectious growth in rice blast disease (24). In *Monascus ruber*, MrHst4 regulates secondary metabolism and citrinin production, an important metabolite in this fungal species (25). These studies collectively demonstrate that Hst3 and Hst4, as members of the sirtuin family, play critical roles in various aspects of fungal biology, including development, metabolism, stress response, and virulence.

In the entomopathogenic fungus *Beauveria bassiana*, which is widely used as a commercial pest control agent (26, 27). The infection begins with conidial spores attaching and germinating on the surface of the insect cuticle, followed by hyphal growth and penetration of the host integument (28, 29). The fungus then produces yeast-like blastospores, which deplete the host’s nutrients in the hemolymph, ultimately leading to the death of the host (30, 31). Four *B. bassiana* HDACs have been proven to play important regulatory roles in this fungal infection process (32–35). Among them, two class I HDACs, BbRpd3 and BbHos2, are known to contribute to sporulation and virulence in *B. bassiana* (32, 33). Moreover, two class III HDACs, BbSir2 and BbHst2, have been previously shown to affect the regulation of carbon and nitrogen metabolism, asexual development, and virulence (34, 35). However, the functions of the remaining sirtuins in *B. bassiana*, namely, BbHst3 and BbHst4, are still unknown. Here, we sought to characterize the contributions of two sirtuins, namely, BbHst3 and BbHst4, in gene transcription, conidiation, cell cycle control, metabolism, stress response, and virulence via characterization of single-gene or double-gene deletion mutants of the *BbHst3* and *BbHst4* genes. Transcriptomic analyses were also conducted to investigate downstream gene targets of BbHst3 and BbHst4 that may be involved in asexual development, cell cycle, and fungus virulence of *B. bassiana*.

## RESULTS

### Bioinformatic features of BbHst3 and BbHst4 and the construction of deletion strains

The BLAST research comparing the yeast Hst3 (GenPept accession no. NP_014668) to *B. bassiana* identified the BbHst3 homolog (NCBI accession code: EJP66006). The nucleotide sequence of BbHst3 is 2,737 bp long and contains two introns. The BbHst3 protein consists of 863 amino acids, with a molecular mass of 94.23 kDa and an isoelectric point of 8.89. It possesses an SIR2 superfamily domain (residues 260–579), which is also found in Hst3 homologs in *S. cerevisiae* and *Fusarium graminearum*, indicating evolutionary conservation (Fig. S1A). Additionally, BbHst3 contains a conserved domain called “PHA03247” (residues 579–856), typically present in large tegument proteins associated with viral entry, suggesting a specific role of Hst3 homologs in entomopathogenic fungi. Overall, BbHst3 shares a high sequence identity of 40%–78% with homologs found in yeasts and other filamentous fungi (Fig. S1B).

Similarly, based on BLAST research with yeast Hst4 (GenPept accession no. NP_010477) as a query, the Hst4 homolog in *B. bassiana* (NCBI accession code: EJP69470) was identified as being encoded by a nucleotide sequence of 1,836 bp containing no introns. The BbHst4 protein consists of 863 amino acids (molecular mass: 66.48 kDa; isoelectric point: 9.37) and contains an SIR2 superfamily domain (residues 144–410), which can also be found in Hst4 homologs in *S. cerevisiae* and *F. graminearum* (Fig. S2A). Overall, BbHst4 shared a 35%–76% high sequence identity with homologs found in yeasts and other filamentous fungi (Fig. S2B). Moreover, the protein length of Hst3 and Hst4 homologs in filamentous pathogens is significantly longer (300–400 amino acids) than that in yeast, suggesting more specific roles for Hst3 and Hst4 in filamentous pathogens.

In order to further investigate the function of *BbHst3* and *BbHst4*, two single-gene deletion mutants (Δ*BbHst3* and Δ*BbHst4*) and a double-gene deletion mutant (Δ*BbHst3*Δ*BbHst4*) were constructed, as detailed in the Materials and Methods. To confirm the function of the *BbHst3* and *BbHst4* genes, the deletion mutants were complemented with the corresponding wild-type genes. All transformants and putative deletion strains or complementation strains were screened by PCR with specific primers, and the gene deletion and complementation were also confirmed by real-time PCR (Fig. S3, primers used are listed in Table S1).

### Role of BbHst3 and BbHst4 in histone H3-K56 acetylation

**FIG 1:**
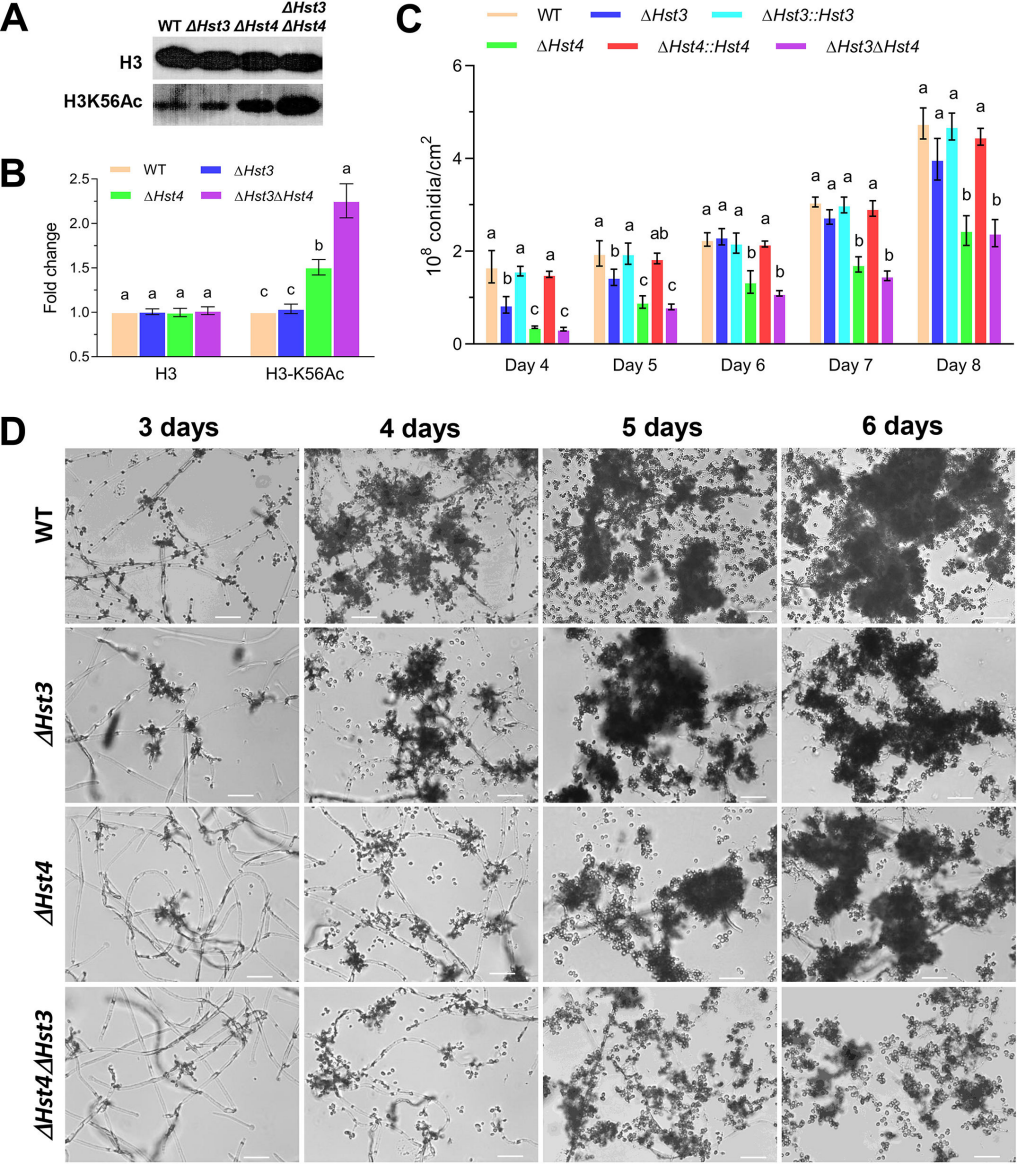
Roles of *B. bassiana* Hst3 and Hst4 homologs in histone H3 deacetylation and sporulation processes. (**A, B**) Western blot analyses detect the level of histone H3-K56 acetylation relative to histone H3 in each mutant strain. (**C**) Conidial yields assessed for each strain over an 8-day time course of growth on Sabouraud dextrose agar (SDAY) plates. (**D**) Microscopic visualization of growing mycelia and sporophores on SDAY plates during 3–6 days post-inoculation (scale bars = 20 µm). Letters indicate a significant difference compared with the wild type (Tukey’s HSD test; *P* < 0.05). Error bars are ±SD from three replicates.

In order to investigate the role of BbHst3 and BbHst4 in mediating acetylation of histone H3 at lysine 56 (H3-K56), Western blotting analysis was performed three times using specific antibodies against histone H3 and histone H3-K56 acetylation, and the signal intensities of all blots were quantified using ImageJ software. As shown in Fig. 1A and B, the loss of BbHst3 had no significant effect on the acetylation of H3-K56 when compared to the wild-type strain. However, the *∆BbHst4* and *∆BbHst3∆BbHst4* strains exhibited enhanced acetylation levels of H3-K56. Specifically, compared to the wild-type, the *∆BbHst4* strain showed an approximately 50% increase in H3-K56 acetylation, while the *∆BbHst3∆BbHst4* strain displayed a more substantial elevation (~2.6-fold) in H3-K56 acetylation levels. These findings, determined through densitometric quantification, suggest that BbHst4 plays a dominant role in mediating the acetylation of histone H3-K56 in *B. bassiana*, while BbHst3 appears to have a subordinate role in this process.

### Contributions of BbHst3 and BbHst4 in asexual development

To assess the impact of BbHst3 and BbHst4 on sporulation, all strains, including the wild-type, the deletion mutants (*∆BbHst3*, *∆BbHst4*, *∆BbHst3∆BbHst4*), and the complemented strains (*∆BbHst3::Hst3*, *∆BbHst4::Hst4*), were grown in rich media [Sabouraud dextrose agar (SDAY)] for a period of 10 days. The conidial yield was examined daily to evaluate the sporulation process. The results, as depicted in Fig. 1C, showed that the *∆BbHst3* strain exhibited a decrease in conidial production compared to the wild-type strain, but the difference was significant only during the 4th and 5th days post-inoculation, with a decrease of approximately 26%–49% (*P <* 0.05). However, from the 6th to the 8th day, no significant differences in conidial production were observed between the *∆BbHst3* strain and the wild-type. In contrast, the *∆BbHst4* strain displayed a dramatic reduction in conidial production. On the 4th day post-inoculation, there was an approximately 80% decrease (*P <* 0.01) in conidial yield compared to the wild-type strain. From the 5th to the 8th day, the *∆BbHst4* strain continued to exhibit a decrease of around 40%–50% (*P <* 0.01) in conidial production. Furthermore, the double-deletion mutant, *∆BbHst3∆BbHst4*, showed an exacerbated deficiency in the sporulation process compared to the wild-type and the *∆BbHst4* strain, although not significant in the latter strain. Similar to the *∆BbHst4* strain, it exhibited a sharp decrease of approximately 80% (*P <* 0.01) in conidial yield on the 4th day. From the 5th to the 8th day, there was a decrease of approximately 50%–60% (*P <* 0.01) compared to the wild-type strain. Additionally, the microscopy observations (Fig. 1D) indicated that the formation of sporophores, which are the ultrastructures for conidiation, was visibly impaired in the *∆BbHst4* and *∆BbHst3∆BbHst4* strains compared to the wild-type strain. Our results also indicated that the sporulation defect caused by gene deletion could be restored by the complementation of the *BbHst3* or *BbHst4* genes, respectively. Taken together, these findings demonstrate that, although the deletion of *BbHst3* had a milder effect on conidial production, BbHst4 has a significant role in sporulation, as its single deletion led to a substantial decrease in conidial production without significant difference compared to the *BbHst3* and *BbHst4* double mutants.

### Impact of BbHst3 and BbHst4 deletions on fungal tolerance to multi-stresses

**FIG 2:**
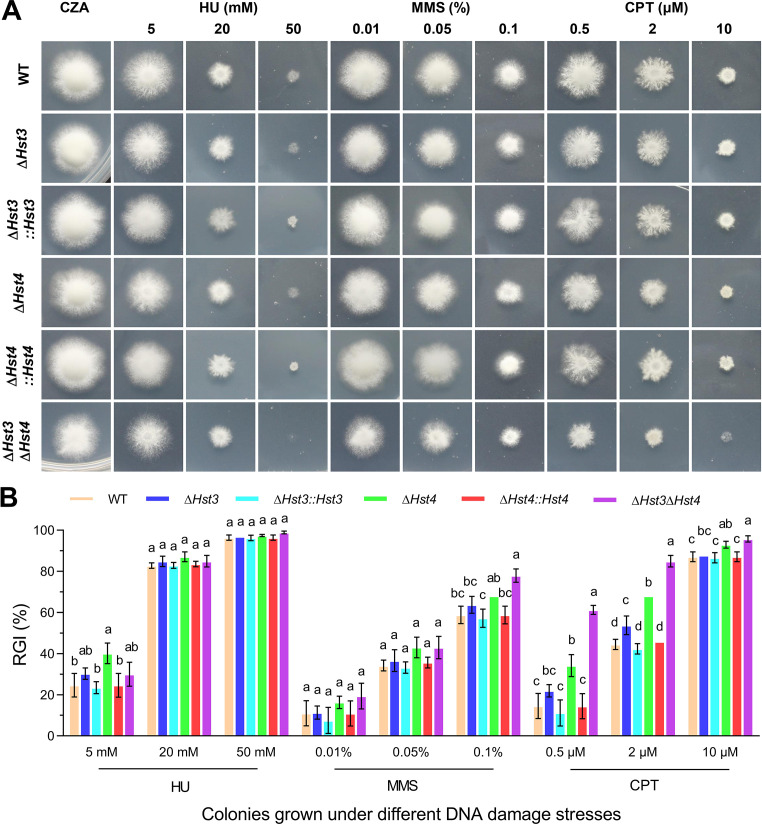
Impact of BbHst3 and/or BbHst4 deletion on fungal tolerance to DNA damage-causing agents. (**A, B**) Representative images and relative growth inhibition index (RGI) of fungal colonies grown at 25°C for 8 days on Czapek-Dox agar supplemented with three different kinds of DNA damage-causing agents: HU (5–50 mM), MMS (0.01%–0.1%), and CPT (0.5–10 µM). All colonies were initiated by spotting 1 µL of 1 × 10^6^ conidia/mL suspension on the plates. All experiments were performed three times. Error bars = ±SD. Letters indicate a significant difference compared with the wild type (Tukey’s HSD test; *P* < 0.05).

To investigate the involvement of BbHst3 and BbHst4 in DNA damage responses, three different DNA damage-causing agents were utilized: hydroxyurea (HU), methyl methanesulfonate (MMS), and camptothecin (CPT). In response to HU, only the deletion of *BbHst4* led to increased sensitivity (~15%, *P <* 0.01) at lower concentrations (5 mM), as shown in Fig. 2A and B. Regarding MMS, both the *∆BbHst4* and *∆BbHst3∆BbHst4* strains displayed enhanced sensitivities (~10% and ~20%, *P <* 0.05) at higher concentrations (0.1%). However, the *∆BbHst3* strain did not exhibit a significant difference in sensitivity to MMS compared to the wild-type strain. Furthermore, all deletion strains showed significantly decreased tolerance to CPT at lower or higher concentrations (0.5–10 µM). For example, the *∆BbHst4* and *∆BbHst3∆BbHst4* strains exhibited reduced tolerance (~40%, *P <* 0.01), while the *∆BbHst3* strain showed a relatively smaller reduction (~10%, *P <* 0.05) at 2 µM CPT. These findings suggest that BbHst3 and BbHst4 are involved in the DNA damage response pathways in *B. bassiana*, with BbHst4 playing a more prominent role in maintaining genomic stability.

**FIG 3:**
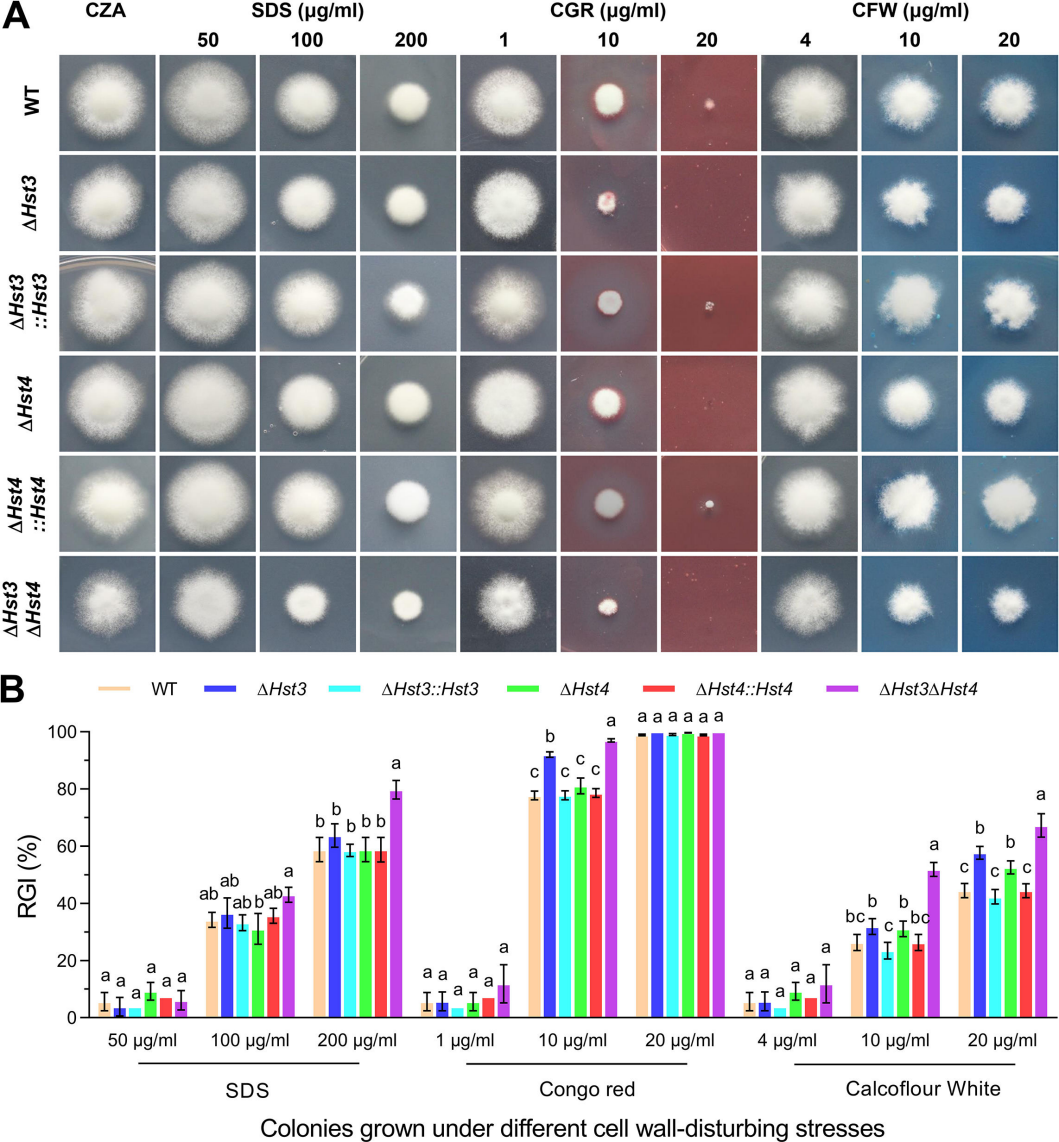
Impact of BbHst3 and/or BbHst4 deletion on fungal tolerance to cell wall-disturbing agents. (**A, B**) Representative images and relative growth inhibition index (RGI) of fungal colonies grown at 25°C for 8 days on Czapek-Dox agar supplemented with three different kinds of DNA damage-causing agents: SDS (50–200 µg mL^−1^), CGR (1–20 µg mL^−1^), and CFW (4–20 µg mL^−1^). All colonies were initiated by spotting 1 µL of 1 × 10^6^ conidia/mL suspension on the plates. All experiments were performed three times. Error bars = ±SD. Letters indicate a significant difference compared with the wild type (Tukey’s HSD test; *P* < 0.05).

To evaluate the impact of *BbHst3* or *BbHst4* deletion on cell wall integrity, three different cell wall-disturbing agents were used: sodium dodecyl sulfate (SDS), Congo red (CGR), and calcofluor white (CFW). In response to SDS, only the *∆BbHst3∆BbHst4* strain exhibited increased sensitivity (~20%, *P <* 0.01) at higher concentrations (200 µg/mL), as shown in Fig. 3A and B. For CGR, both the *∆BbHst3* and *∆BbHst3∆BbHst4* strains displayed enhanced sensitivities (~15%, ~20%, *P <* 0.01) at middle concentrations (10 µg/mL). However, the *∆BbHst4* strain did not show a significant difference in sensitivity to CGR compared to the wild-type strain. Furthermore, all deletion strains showed significantly decreased tolerance to CFW at higher concentrations (20 µg/mL). The reductions in tolerance ranged from 8% to 20% (*P <* 0.05) for all deletion strains. Additionally, the *∆BbHst3∆BbHst4* strain exhibited increased sensitivity (~25%, *P <* 0.01) at middle concentrations (10 µg/mL) of CFW. These results suggest that the deletion of *BbHst3* and/or *BbHst4* affects cell wall integrity in *B. bassiana*.

The impact of *BbHst3* or *BbHst4* deletion on osmotic and oxidative stress responses was also investigated using osmotic stresses (NaCl, KCl, sorbitol) and oxidative stresses (H_2_O_2_, menadione). In response to osmotic stresses, the *∆BbHst3∆BbHst4* strain displayed elevated sensitivity (~15%, *P <* 0.05) to NaCl and KCl, as depicted in Fig. S4A and B. Additionally, the *∆BbHst4* strain exhibited increased sensitivity (~13%, *P <* 0.05) to NaCl. Regarding oxidative stresses, all mutant strains exhibited significantly reduced tolerance (~10%-20%, *P <* 0.05) to H_2_O_2_. This indicates that the deletion of either *BbHst3* or *BbHst4*, or both, compromises the ability of *B. bassiana* to withstand oxidative stress caused by H_2_O_2_. Furthermore, only the *∆BbHst3∆BbHst4* strain showed reduced tolerance (15%, *P <* 0.05) to menadione, another oxidative stress-inducing agent. These findings demonstrate that BbHst3 and BbHst4 play important roles in the response to osmotic and oxidative stresses in *B. bassiana*. All of these phenotypic defects in tolerating multi-stresses due to gene deletion were restored in the complemented strains.

### Contribution of *BbHst3* and *BbHst4* to *B. bassiana* virulence

**FIG 4:**
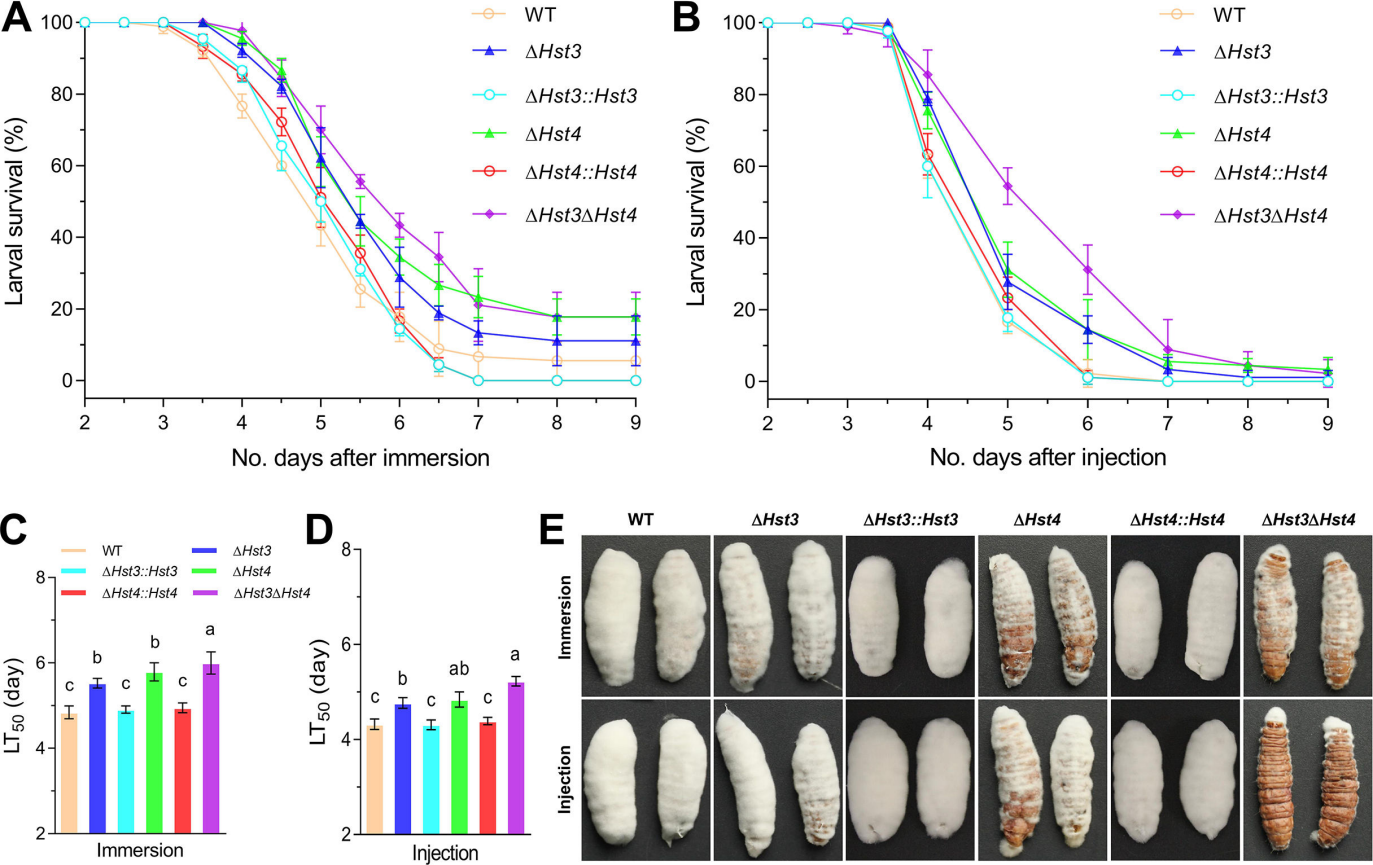
Contribution of BbHst3 and/or BbHst4 to *B. bassiana* virulence and virulence-related properties. (**A, B**) Insect bioassay survival curves using the greater wax moth (*G. mellonella*) larvae as hosts after topical application (immersion) and intra-hemocoel injection, respectively. (**C, D**) The median lethal time to kill 50% of the target hosts (LT_50_) for each strain in topical application and intra-hemocoel injection bioassays. (**E**) Representative images of fungal outgrowth on the surfaces of cadavers 4 days post-death after being killed by each strain. All experiments were performed three times. Error bars = ±SD. Letters indicate a significant difference compared with the wild type (Tukey’s HSD test, *P* < 0.05).

To assess the contribution of BbHst3 and BbHst4 to the pathogenicity of *B. bassiana*, insect bioassays were conducted using *Galleria mellonella* larvae as the host. Both topical application and intra-hemocoel injection methods were employed in these assays. In topical bioassays, the median lethal time to kill 50% of the target hosts (LT_50_) for the wild-type strain was determined to be 4.84 ± 0.12 days (Fig. 4A and C). In comparison, the *∆BbHst3* strain, *∆BbHst4* strain, and *∆BbHst3∆BbHst4* strain exhibited increased LT_50_ values of 5.53 ± 0.09 days (~14% increase, *P <* 0.05), 5.80 ± 0.17 days (~20% increase, *P <* 0.01), and 6.00 ± 0.21 days (~24% increase, *P <* 0.01), respectively. In the intra-hemocoel injection assays, similar trends were observed. The LT_50_ value for the wild-type strain was 4.32 ± 0.09 days (Fig. 4B and D). In contrast, the *∆BbHst3* strain, *∆BbHst4* strain, and *∆BbHst3∆BbHst4* strain showed increased LT_50_ values of 4.77 ± 0.09 days (~10% increase, *P <* 0.01), 4.84 ± 0.13 days (~12% increase, *P <* 0.01), and 5.23 ± 0.08 days (~21% increase, *P <* 0.01), respectively. These results indicate that the deletion of *BbHst3*, *BbHst4*, or both genes prolongs the median lethal time, suggesting a decrease in pathogenicity, while the complemented strains could regain their full pathogenicity upon insect host.

Additionally, after 4 days post-death, the fungal outgrowths observed on the cadavers of the *∆BbHst4* and *∆BbHst3∆BbHst4* strains were noticeably reduced compared to the control strains (Fig. 4E). This observation suggests that the absence of *BbHst4* and/or *BbHst3* affects fungal proliferation and colonization in insect cadavers. Overall, the results from the insect bioassays indicate that BbHst3 and BbHst4 are important for the pathogenicity of *B. bassiana*. Their deletion results in prolonged median lethal times and reduced fungal outgrowth on the insect cadavers, underscoring their contributions to successful infection and colonization.

### Global regulatory role of BbHst3 and BbHst4 in gene transcription

**FIG 5:**
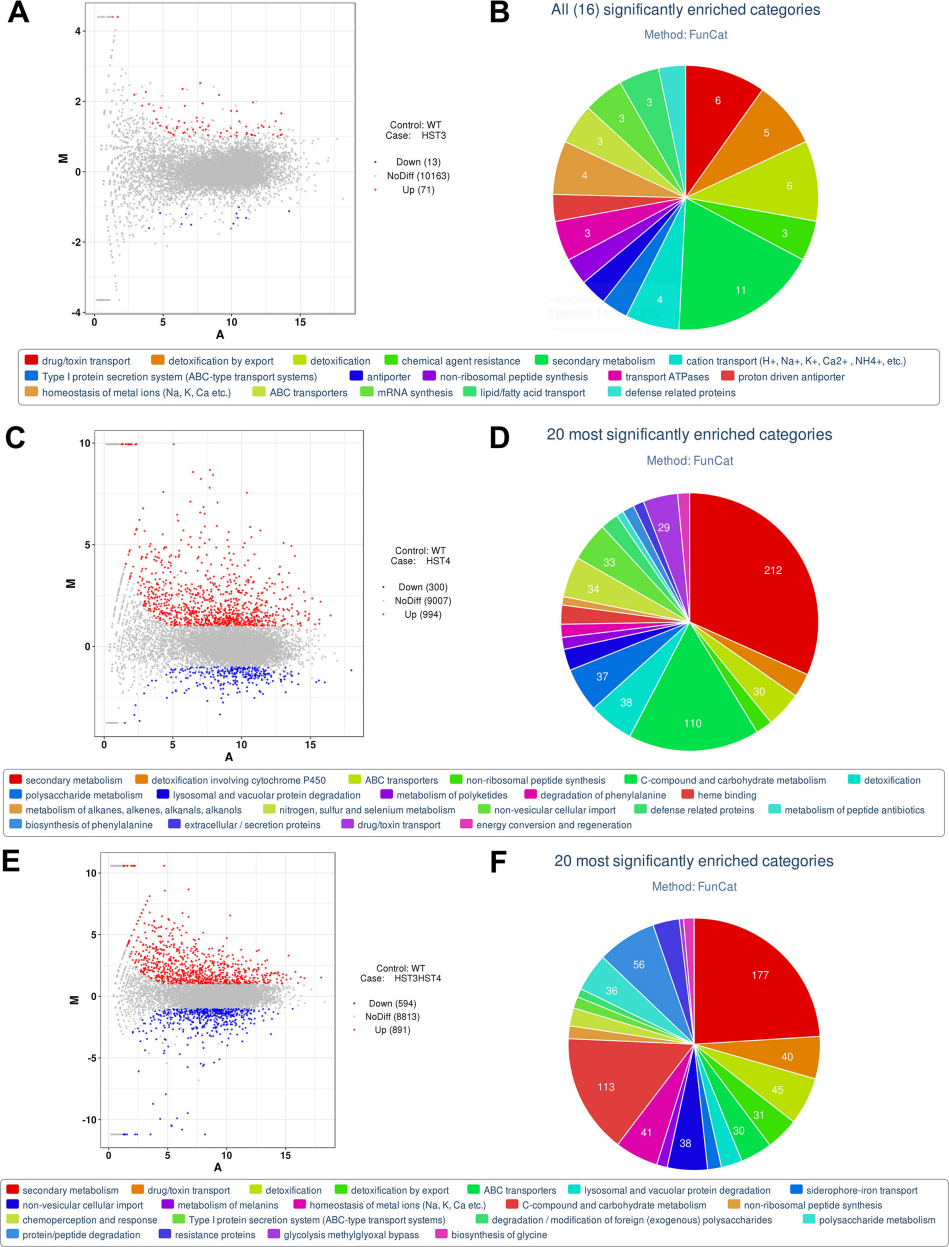
Comparative transcriptomic analyses of Δ*BbHst3*, Δ*BbHst4*, and Δ*BbHst3*Δ*BbHst4* strains and wild type. (**A, C, E**) MA-plot analysis of the genes significantly upregulated, downregulated, and not differentially regulated (No-Diff) in Δ*BbHst3*, Δ*BbHst4*, and Δ*BbHst3*Δ*BbHst4* versus wild type, respectively. (**B, D, F**) FunCat annotation into different functional categories of significantly regulated genes in Δ*BbHst3*, Δ*BbHst4*, and Δ*BbHst3*Δ*BbHst4* versus wild type, respectively.

To gain insight into the global gene networks affected by the loss of *BbHst3* and *BbHst4*, comparative transcriptomic analyses were conducted. These analyses involved the mutant strains (*∆BbHst3*, *∆BbHst4*, and *∆BbHst3∆BbHst4*) and the wild-type strain of *B. bassiana*. In the *∆BbHst3* strain, a total of 84 differentially expressed genes (DEGs) were identified compared to the wild-type strain. Among these DEGs, 13 genes were downregulated [Log_2_ (ratio): −1.61 to −1.00], while 71 genes were upregulated [Log_2_ (ratio): +1.00 to +4.40] (Fig. 5A; Table S2). For the *∆BbHst4* strain, a total of 1,294 DEGs were identified, including 300 downregulated genes [Log_2_ (ratio): −3.66 to −1.00] and 994 upregulated genes [Log_2_ (ratio): +1.00 to +9.94] (Fig. 5C; Table S2). Furthermore, the deletion of both *BbHst3* and *BbHst4* resulted in altered expression of 1,485 genes, including 594 downregulated genes [Log_2_ (ratio): −11.20 to −1.00] and 891 upregulated genes [Log_2_ (ratio): +1.00 to +10.58] (Fig. 5E; Table S2). These findings indicate that the loss of *BbHst3* and/or *BbHst4* has a significant impact on the global gene expression profile of *B. bassiana*.

To further analyze the DEGs in each group, FunCat category annotation was applied. In the *∆BbHst3* strain, a total of 22 DEGs were classified into 16 functional classes (Fig. 5B; Table S3). These functional classes included secondary metabolism (11), detoxification (6), drug/toxin transport (6), detoxification by export (5), cation transport (4), homeostasis of metal ions (4), and others. Among the DEGs in the *∆BbHst3* strain, two ABC transporters and three MFS multidrug transporters were found to be differentially regulated. These transporters play crucial roles in the efflux of various molecules, including drugs and toxins, and contribute to detoxification processes (36). Additionally, several other types of transporters were identified, such as a low-affinity iron transporter, a mitochondrial CorA family metal ion transporter, and a vacuolar calcium ion transporter. The identification of differentially regulated transporters and genes involved in secondary metabolism highlights the impact of *BbHst3* deletion on cellular processes related to detoxification, ion transport, and secondary metabolite production.

In the *∆BbHst4* strain, a total of 486 DEGs were classified into 59 functional classes (Fig. 5D; Table S4). Among these classes, the 20 most significantly enriched categories included secondary metabolism (212), C-compound and carbohydrate metabolism (110), detoxification, polysaccharide metabolism (37), nitrogen, sulfur, and selenium metabolism (34), non-vesicular cellular import (33), ABC transporters (30), drug/toxin transport (29), and others. Various types of transporters were found to be differentially regulated in the *∆BbHst4* strain. This includes 19 ABC transporters, 31 MFS multidrug transporters, 4 OPT oligopeptide transporters, and 3 sugar transporters. Regarding detoxification, several genes showed altered expression in the *∆BbHst4* strain. This includes 30 cytochrome P450s, 10 FAD-binding/dependent proteins, 9 flavin-binding/containing monooxygenases, and 12 short-chain dehydrogenases. Importantly, a large array of DEGs in the *∆BbHst4* strain are related to cell rescue, defense, and virulence. This includes six subtilase/subtilisin-like proteins, six polyketide synthases, six peptidases, three chitinases, two lipases, and one cuticle-degrading protease. These genes are associated with processes such as host colonization, nutrient acquisition, and evasion of host defenses (28). These findings highlight the extensive impact of *BbHst4* deletion on gene expression patterns, with implications for secondary metabolism, cellular metabolism, transport processes, detoxification, and the regulation of cell rescue, defense, and virulence-related genes.

In the *∆BbHst3∆BbHst4* strain, a total of 532 DEGs were classified into 53 functional classes (Fig. 5F; Table S5). The 20 most significantly enriched categories among these DEGs included secondary metabolism (177), C-compound and carbohydrate metabolism (113), protein/peptide degradation (38), detoxification (39), homeostasis of metal ions (Na, K, Ca, etc.) (40), drug/toxin transport (41), non-vesicular cellular import, polysaccharide metabolism (36), detoxification by export (31), ABC transporters (30), and others. A wide range of transporters were found to be differentially regulated in the *∆BbHst3∆BbHst4* strain. This includes 17 ABC transporters, 33 MFS multidrug transporters, 6 oligopeptide transporters, 5 iron transporters, 4 hexose transporters, and 3 sugar transporters. In terms of protein/peptide degradation, 30 different peptidases and 17 different proteases showed altered expression in the *∆BbHst3∆BbHst4* strain. These include carboxypeptidases, peptidase family A4/M3/S8, serine peptidases, tripeptidyl peptidases, alkaline serine proteases, metalloproteases, protease S8 tripeptidyl peptidases, trypsin-like proteases, aspartic proteases, and subtilisin-like proteases. This suggests that the absence of both *BbHst3* and *BbHst4* affects protein and peptide degradation processes in *B. bassiana*. Furthermore, the *∆BbHst3∆BbHst4* strain exhibited altered expression of genes involved in detoxification processes. This includes 24 cytochrome P450s, 9 FAD binding/dependent proteins, 7 flavin-binding/containing monooxygenases, and 11 short-chain dehydrogenases. Additionally, several lipases and chitinases involved in cuticle degradation were differentially regulated, including six lipases and five chitinases. This suggests that the absence of BbHst3 and BbHst4 affects the breakdown of the insect cuticle, which is crucial for fungal infection. Interestingly, there were also differentially expressed genes encoding fungal infection-related specific proteins or factors in the *∆BbHst3∆BbHst4* strain. This includes four fungal-specific transcription factors, four toxin proteins, three polyketide synthases, and two SCP-like extracellular proteins. Taken together, the analysis of DEGs in the *∆BbHst3∆BbHst4* strain reveals significant alterations in transport processes, protein/peptide degradation, detoxification, cuticle degradation, and the expression of fungal infection-related factors.

**FIG 6:**
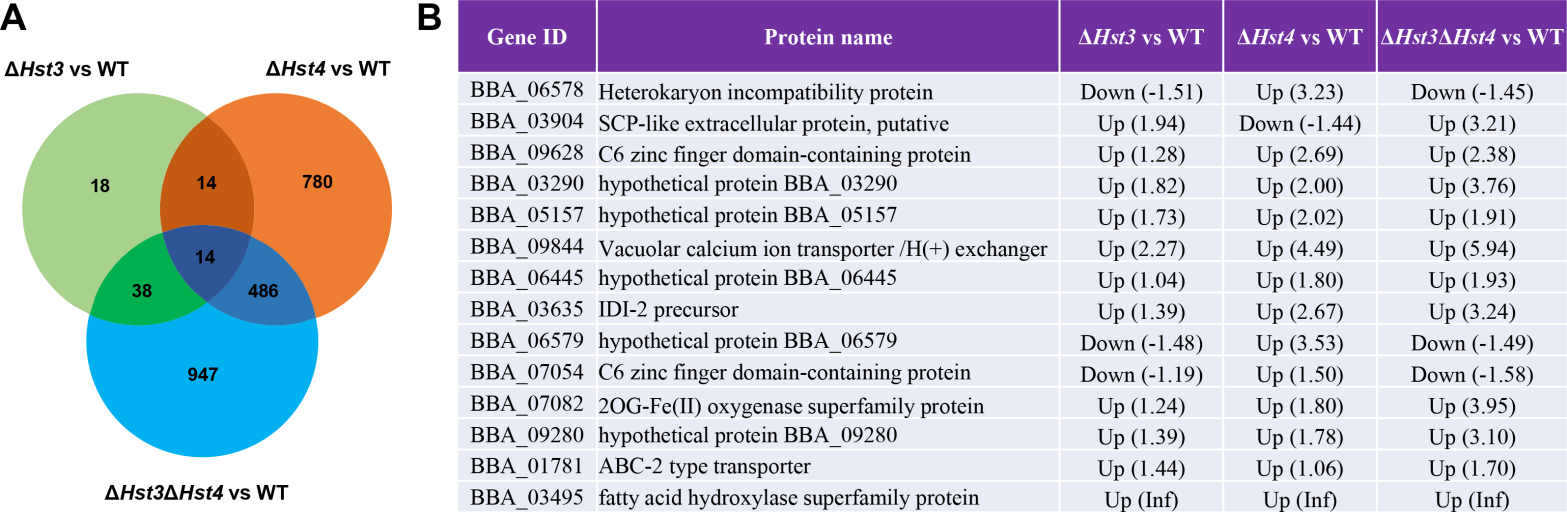
(**A**) Venograms showing the numbers of shared DEGs in each group. (**B**) The differential regulation of 14 shared DEGs in Δ*BbHst3*, Δ*BbHst4*, and Δ*BbHst3*Δ*BbHst4* versus wild type, respectively.

The comparison of DEGs among the mutant strains revealed interesting patterns (Fig. 6A). The *∆BbHst4* strain and the *∆BbHst3∆BbHst4* strain shared a larger number of DEGs, with 486 genes in common. These shared DEGs accounted for 37.6% and 32.7% of the total number of DEGs in each respective group. On the other hand, the *∆BbHst3* strain and the *∆BbHst3∆BbHst4* strain only showed 38 shared DEGs, while the *∆BbHst3* strain and the *∆BbHst4* strain only showed 14 shared DEGs. Among the 14 shared DEGs in all three groups, most of them showed upregulated expression, indicating a consistent role of BbHst3 and BbHst4 as gene repressors (Fig. 6B). Two transporters, including an ABC-2 type transporter and a vacuolar calcium ion transporter, were consistently upregulated in all three mutant strains. Furthermore, two C6 zinc finger domain-containing proteins and an SCP-like extracellular protein showed altered expression in all three mutant strains. Additionally, two metabolic enzymes, including an IDI-2 precursor and a fatty acid hydroxylase, were consistently upregulated. Moreover, five uncharacterized proteins were identified to be differentially regulated in all mutant strains, with most showing upregulated expression. These proteins represent potential targets co-regulated by BbHst3 and BbHst4, highlighting the complexity of their regulatory functions and the possibility of novel regulatory pathways. Overall, the comparison of shared DEGs among the mutant strains reveals both overlapping and distinct regulatory effects of BbHst3 and BbHst4 in transport processes and cellular metabolism.

## DISCUSSION

Sirtuins have been revealed to play important roles in multiple cellular processes and pathogenicity in various filamentous pathogens, such as human pathogenic fungi (*C. albicans*, *C. neoformans*) and plant pathogenic fungi (*Magnaporthe oryzae*, *Ustilago maydis*) (20, 37–39). In *B. bassiana*, the two sirtuins BbSir2 and BbHst2 have been shown to be involved in asexual development, carbon/nitrogen utilization, stress tolerance, and fungal pathogenicity (34, 35). The deletion of BbSir2 resulted in significant changes in protein expression and lysine acetylation patterns, highlighting its regulatory functions at the post-translational level (34). Similarly, the deletion of BbHst2 led to differential expression of numerous genes, indicating its role in transcriptional regulation (35). In this study, the characterization of BbHst3 and BbHst4 further expands our understanding of the sirtuin family in *B. bassiana*. These two sirtuins were found to be important for sporulation, cell wall integrity, DNA damage repair, and fungal virulence. The observed phenotypic changes in the mutant strains highlight the specific contributions of BbHst3 and BbHst4 to these cellular processes. According to the transcriptomic analyses, although the deletion of *BbHst3* had a minor impact on global gene transcription, the single deletion of *BbHst4* and the double deletion of *BbHst3* and *BbHst4* resulted in a large number of deregulated genes, of which 37.6% and 32.7% in each respective group were shared. That means there are 62.4% and 67.3% of DEGs different between the *BbHst4* mutant and the *BbHst3* and *BbHst4* double mutants. This interesting finding suggests that the deletion of *BbHst4* may have a different impact on the wild-type and the *BbHst3* mutant. The reasons might be that (i) the deletion of *BbHst3* resulted in the deregulation of some genes, providing a more complicated background for the *BbHst4* deletion process; (ii) there are still lots of unknown relationships between BbHst3 and BbHst4 that contribute to the difference. Overall, the transcriptomic analyses revealed the global regulatory roles of BbHst3 and BbHst4 in gene transcription, affecting the expression of numerous genes involved in diverse functional categories.

Conidiation is a critical process for the production of conidia, which are the active ingredients used in insect biological control applications using *B. bassiana* (40). In previous studies on other filamentous fungi, key regulatory genes involved in conidiation have been identified, including AbaA, BrlA, and WetA, as well as upstream pathway components such as FlbA, FlbB, FlbC, FlbD, and FlbE and downstream regulators like VosA and WetA (41, 42). Mutation of either the *BrlA*, *AbaA*, or *WetA* genes resulted in severely impairing asexual development and conidiation in *B. bassiana* (43, 44). Our data show that the deletion of *BbHst3* has no significant impact on conidiation yield, while the deletion of *BbHst4* and the double deletion of *BbHst3* and *BbHst4* result in a significant reduction in conidiation by approximately 50%. This reduction in conidiation is consistent with the decreased expression of development-related genes observed in the global transcriptomic analyses. Specifically, in the Δ*BbHst4* strain, the transcription of *FlbA*, *FlbD*, *BrlA*, *AbaA*, and *WetA* was significantly downregulated, which may explain the impaired conidial production in this strain. Furthermore, the differential regulation of hydrophobic proteins, including five hydrophobins (Hyd1-5) and adhesin proteins, in the Δ*BbHst4* and Δ*BbHst3*Δ*BbHst4* strains may contribute to the observed reduction in conidiation. These proteins are known to play important roles in conidial development and adhesion (45, 46). The conserved role of Hst3 and Hst4 in regulating asexual development, as demonstrated by impaired growth or conidiation in other filamentous fungi (*Aspergillus* spp., *M. oryzae*, etc.) (21–24), further supports the importance of these sirtuins in conidiation processes across different fungal species. Overall, our findings provide valuable insights into the regulatory mechanisms of conidiation and highlight the specific contributions of BbHst3 and BbHst4 in this essential fungal developmental process.

The ability of conidia to tolerate various stressors and complicated environments is also critical for the biological potential of fungal strains (47). Histone H3-K56 acetylation is known to be crucial for DNA damage repair and genome integrity (13), and the deacetylation of H3-K56 is mediated by Hst3 and Hst4 (10, 11). In our study, the deletion of *BbHst4* or the double deletion of *BbHst3* and *BbHst4* leads to a significant increase in the level of H3-K56 acetylation, while the single deletion of *BbHst3* has no significant impact on H3-K56 acetylation. This is consistent with the known role of Hst3 and Hst4 as deacetylases of H3-K56. Furthermore, the increased H3-K56 acetylation in the Δ*BbHst4* and Δ*BbHst3*Δ*BbHst4* strains is associated with reduced tolerance to DNA damage-causing agents, such as CPT, MMS, and HU. This suggests that BbHst4 and, to a lesser extent, BbHst3 play important roles in maintaining DNA integrity and responding to DNA damage stresses. The downregulation of a DNA photolyase gene (Phr2, required for ultraviolet DNA damage repair) (48) and a histone synthetic lethal gene (Hsl1, essential for cell cycle checkpoint) (49) in the Δ*BbHst4* strain may contribute to the disturbed H3-K56 acetylation and the reduced tolerance to DNA damage.

In terms of cell wall-disturbing stresses, the deletion of *BbHst3* appears to play a more predominant role than that of *BbHst4*. The Δ*BbHst3* and Δ*BbHst3*Δ*BbHst4* strains showed increased sensitivity to cell wall-disturbing agents such as SDS, Congo red, and calcofluor white, while the Δ*BbHst4* strain only showed increased sensitivity to calcofluor white at higher concentrations. This suggests that BbHst3 is more involved in maintaining cell wall integrity in response to these stresses. Consistent with these altered phenotypes, the transcription of five WSC domain-containing proteins (50) was significantly regulated in the Δ*BbHst4* and Δ*BbHst3*Δ*BbHst4* strains. Also, three catalases and one superoxide dismutase (51) showed differential expression in these mutant strains, which may account for the enhanced sensitivity to H_2_O_2_ and menadione. The altered phenotypes and transcriptional changes observed in the mutant strains provide valuable insights into the specific roles of BbHst3 and BbHst4 in histone acetylation, DNA damage repair, and stress tolerance in *B. bassiana*.

Our data provide compelling evidence for the crucial role of BbHst3 and BbHst4 in the virulence of *B. bassiana* against insect hosts. The deletion of either *BbHst3* or *BbHst4*, as well as the double deletion of both genes, resulted in a significant reduction in the ability of *B. bassiana* to kill insect hosts through topical application and cuticle-penetrating injection. The reduced virulence observed in the mutant strains is consistent with the downregulation or differential regulation of several key virulence factors involved in insect cuticular degradation and fungal toxin synthesis. This includes subtilisin-like proteases (Pr1A, Pr1C, Pr1F, Pr1G) and cuticle-degrading proteases (CDEP1, CDEP2) that are essential for fungal penetration of the insect cuticle (52). Furthermore, the expression of six polyketide synthases and four oosporein synthases involved in fungal toxin synthesis was also altered in the mutant strains (53, 54). These changes in gene expression likely contribute to the decreased ability of the *BbHst3*/*BbHst4* deletion strains to infect and kill insect hosts. The differential regulation of LysM domain-containing proteins, such as LysM1, LysM2, LysM5, LysM6, and LysM7, in the Δ*BbHst4* and Δ*BbHst3*Δ*BbHst4* strains is also noteworthy. The LysM domain-containing proteins are involved in various biological processes, including cell wall degradation and the recognition of chitin, an important component of fungal cell walls (55). Their altered expression suggests potential disruptions in cell wall integrity and host-fungus interactions in the mutant strains. The impaired virulence observed in the Δ*BbHst3* and Δ*BbHst4* strains, as well as the Δ*BbHst3*Δ*BbHst4* strain, is consistent with findings in other fungal pathogens such as *C. albicans*, *C. neoformans*, and *M. oryzae* (19, 20, 24), where the deletion of *Hst3* or *Hst4* also results in reduced pathogenicity. This highlights the important role of Hst3 and Hst4 in regulating fungal virulence across different fungal species and hosts.

Overall, our study suggests that BbHst3 and BbHst4 play important roles in various aspects of *B. bassiana*’s biology and pathogenicity. Their deletion leads to impaired sporulation, decreased tolerance to DNA damage stress and cell wall perturbing agents, and reduced virulence. These effects can be attributed to a combination of decreased tolerance to stresses and the differential expression of genes involved in development, stress response, and virulence. The findings highlight the regulatory functions of BbHst3 and BbHst4 in coordinating gene expression and cellular processes in *B. bassiana*.

## MATERIALS AND METHODS

### Strains and culturing conditions

The wild-type strain of *B. bassiana* ARSEF 2860 (WT) was cultured on SDAY containing 4% glucose, 1% peptone, 1% yeast extract, and 1.5% agar. *Escherichia coli* DH5a was grown in Luria-Bertani medium for plasmid propagation, supplemented with appropriate antibiotics for selection. *Agrobacterium tumefaciens* AGL-1 was cultured in yeast extract broth (0.5% sucrose, 1% peptone, 0.1% yeast extract, and 0.05% MgSO_4_). For specific experiments, Czapek-Dox agar (CZA) was used as a defined medium, consisting of 3% sucrose, 0.3% NaNO_3_, 0.05% KCl, 0.1% K_2_HPO_4_, 0.05% MgSO_4_, 0.001% FeSO_4_, and 1.5% agar.

### Bioinformatic analysis of Hst3 and Hst4 homologs in *B. bassiana*

To identify the *B. bassiana* Hst3 and Hst4 homologs, the amino acid sequences of *S. cerevisiae* Hst3 (NP_014668) and Hst4 (NP_010477) were used as queries to search the *B. bassiana* ARSEF 2860 genomic database (NCBI accession: NZ_ADAH00000000). The resulting sequences were then compared to sequences from other representative fungi using the NCBI database (http://blast.ncbi.nlm.nih.gov/) to identify homologous sequences. To predict the conserved domains within the identified sequences, the SMART program (http://smart.embl-heidelberg.de) was used. This allowed for the identification of conserved domains shared by Hst3 and Hst4 across different fungal species. Phylogenetic analysis was also performed using the MEGA7 software (http://www.megasoftware.net) to determine the evolutionary relationships between the identified Hst3 and Hst4 homologs in different fungi. The molecular weight and isoelectric point of BbHst3 and BbHst4 were predicted using the ExPASy-Compute pI/Mw tool (https://web.expasy.org/compute_pi/) based on the amino acid sequence composition of the proteins.

### Generation of *B. bassiana* Hst3 and Hst4 deletion/complementation strains

The single gene-knockout strains, Δ*BbHst3* and Δ*BbHst4*, were generated using homologous recombination techniques. In this process, the selection markers *sur* or *bar*, which confer resistance to chlorimuron ethyl or phosphinothricin, were used. The detailed methodology for constructing these knockout strains can be found in a previous study (34). To generate the double mutant strain Δ*BbHst3*Δ*BbHst4*, the *BbHst4* gene was deleted in the Δ*BbHst3* strain. The *bar* gene was used as the selection marker in this step. The complementation of BbHst3 or BbHst4 was conducted by ectopic integration of a cassette consisting of its full-length sequence in the *BbHst3*/*BbHst4* deletion mutant, and a *bar* or *sur* marker was used in the screening step. Putative transformants were screened using either chlorimuron ethyl (10 µg mL^−1^) or phosphinothricin (200 µg mL^−1^) to select for the deletion/complementation strains, depending on the selection marker used. Following selection, the transformants were single-spore purified to obtain homogenous cultures. To confirm the gene deletion/complementation, PCR and real-time PCR were performed using specific primers. The primers used in the study can be found in Table S1.

### Western blotting for detecting histone H3-K56 acetylation

To assess the protein levels of histone H3 and the acetylation of H3-K56, the strains (wild-type, Δ*BbHst3*, Δ*BbHst4*, and Δ*BbHst3*Δ*BbHst4*) were cultured in Sabouraud dextrose broth liquid medium with aeration for 3 days at 25°C. Total protein was then extracted from the cultures, and the protein concentrations were then determined using the BCA Protein Assay Kit (Thermo Fisher Scientific). For Western blotting, the extracted proteins were separated by gel electrophoresis and transferred onto a membrane. The membrane was then probed with an anti-histone H3 antibody and an H3-K56Ac antibody. The anti-histone H3 antibody used was from Abcam (catalog # ab1791), and the H3-K56Ac antibody was from Merck Millipore (catalog # 06-599). After incubation with the primary antibodies, the membrane was washed and incubated with a secondary antibody, specifically goat anti-rabbit IgG antibody (Boster, Wuhan, China). Chemiluminescence was used to detect the protein bands using a suitable detection kit (Amersham Biosciences, Shanghai, China). The experiments were repeated three times for each strain. The intensity of the protein bands on the blots was quantified using ImageJ software, which can be accessed at https://imagej.nih.gov/ij/.

### Investigation of sporulation and stress tolerance

To assess conidial production, a suspension of 1 × 10^7^ conidia/mL was prepared. Then, 100 µL of the conidial suspension was spread onto SDAY plates (Sabouraud dextrose agar supplemented with yeast extract). The plates were incubated at 25°C under a light-dark cycle of 12 hours light and 12 hours dark. Starting from day 4 and continuing until day 9, three 5-mm plugs were taken daily from each plate. These plugs were then placed in 1 mL of a 0.02% Tween 80 solution and subjected to mild ultrasonication to disperse the conidia. The total number of conidia was determined by counting them using a hemocytometer. This procedure was performed for each strain, including the wild-type, Δ*BbHst3*, Δ*BbHst4*, and Δ*BbHst3*Δ*BbHst4*, to compare their conidial production over time.

To assess stress responses, a suspension of 1 × 10^6^ conidia/mL was prepared. Then, 1 µL of the conidial suspension was spotted onto CZA plates or CZA-modified plates containing different stress-inducing agents. The CZA-modified plates were prepared by incorporating specific stress-causing agents into the agar at the following concentrations: (i) three osmotic stress-causing agents: 0.4 M NaCl, 0.4 M KCl, or 0.6 M sorbitol; (ii) two oxidative stress-causing agents: 2 mM H_2_O_2_ or 0.02 mM menadione; (iii) three cell wall-perturbing agents: 50–200 μg mL^−1^ SDS, 1–20 μg mL^−1^ Congo red or 4–20 μg mL^−1^ calcofluor white; (iv) three DNA damage-causing agents: 5–50 mM HU, 0.01%-0.1% MMS, or 0.5–10 μM CPT. After spotting the conidial suspension, the plates were incubated at 25°C for 8 days. The diameter of each colony was measured, and the relative growth inhibition (RGI) index was calculated using the formula: RGI = (Sc − Ss)/Sc × 100, where Sc represents the area of the control colony and Ss represents the area of the stressed colony. The RGI index provides a measure of the growth inhibition caused by a specific stress condition.

### Bioassays using *Gellaria mellonella*

For the insect bioassays, larvae of *Galleria mellonella* (Pyralidae) were used to assess the virulence of the fungal strains. Two methods—topical application and intra-hemocoel injection—were employed. (i) In the topical application method, approximately 30 larvae were immersed in a 30 mL suspension of *B. bassiana* conidia at a concentration of 1 × 10^7^ conidia/mL for 10 seconds. (ii) In the intra-hemocoel injection method, each larva was injected with 5 µL of a suspension containing *B. bassiana* conidia at a concentration of 1 × 10^5^ conidia/mL. The injection was performed directly into the hemocoel. After immersion or injection, the larvae were placed in a suitable environment at 25°C and monitored every 12 hours for a period of 10 days. Larval mortality was recorded during the observation period. The experiments were repeated three times to ensure reliable results. The mean lethal time to kill 50% of the larvae (LT_50_) was estimated using probit analysis. Additionally, after 4 days post-death, fungal hyphae growing on the surfaces of dead larvae were observed and examined.

### Transcriptomic analysis

In the transcriptomic analyses, three replicates of each *B. bassiana* strain, including the Δ*BbHst3*, Δ*BbHst4*, Δ*BbHst3*Δ*BbHst4*, and wild-type strains (12 samples total), were grown on SDAY plates overlaid with cellophane for 4 days. After the incubation period, fungal cells were collected, and total RNA was extracted using RNA Trizol. The mRNA fraction was isolated using magnetic oligo (dT) beads, and the mRNAs were fragmented by ionic disruption. First-strand cDNA synthesis was performed using random hexamer primers, followed by second-strand cDNA synthesis using a cDNA synthesis kit. The cDNA libraries were constructed with end-labeled adaptors after purification, end-repair, and single adenine addition. The constructed cDNA libraries were subjected to sequencing using an Illumina HiSeq platform. The resulting sequencing reads, or tags, were filtered and aligned with the *B. bassiana* ARSEF 2860 genome (56). Transcripts were considered significantly regulated if the Log_2_ (Δ*BbHst3*/WT ratio) was less than −1 (indicating downregulation) or greater than 1 (indicating upregulation) with a false discovery rate less than 0.01. The data were then normalized as fragments per kilobase of exon per million fragments mapped. Functional annotation of all identified DEGs was performed using FunCat category classification (https://elbe.hki-jena.de/fungifun/).

## Data Availability

The transcriptomics data generated in this study have been deposited in the Sequence Read Archive (SRA) on NCBI (https://www.ncbi.nlm.nih.gov/) with the data set identifier PRJNA721731.

## SUPPLEMENTAL MATERIAL

Supplemental material spectrum.03137-23-s0001.pdf Fig. S1 to S4; Table S1.

Supplemental tables spectrum.03137-23-s0002.xlsx Tables S2 to S5.

ASM does not own the copyrights to Supplemental Material that may be linked to, or accessed through, an article. The authors have granted ASM a non-exclusive, world-wide license to publish the Supplemental Material files. Please contact the corresponding author directly for reuse.
