# Supplementary material for: Two sirtuin proteins, Hst3 and Hst4, modulate asexual development, stress tolerance, and virulence by affecting global gene expression in Beauveria bassiana
Source: Microbiol Spectr. 2024 Jan 9;12(2):e03137-23. doi: 10.1128/spectrum.03137-23 (PMC10846017; doi:10.1128/spectrum.03137-23)
Supplement: Supplemental material — Fig. S1 to S4; Table S1. [file spectrum.03137-23-s0001.pdf]

Supporting Information

Running title: Role of two sirtuin proteins Hst3 and Hst4 in *B. bassiana*

Two sirtuin proteins Hst3 and Hst4 modulate asexual development, stress tolerance and virulence via affecting global gene expression in *Beauveria bassiana*

Qing Cai,[a*] Li Tian,[b] Jia-Tao Xie,[a] Dao-Hong Jiang[a]

[a] College of Plant Science and Technology, State Key Laboratory of Agricultural Microbiology, Huazhong Agricultural University, Wuhan, Hubei, 430070, China

[b] Shandong Provincial Key Laboratory of Microbial Engineering, Department of Bioengineering, Qilu University of Technology, Jinan, Shandong, 250022, China

* Corresponding author: Qing Cai, E-mail: caiqing@mail.hzau.edu.cn

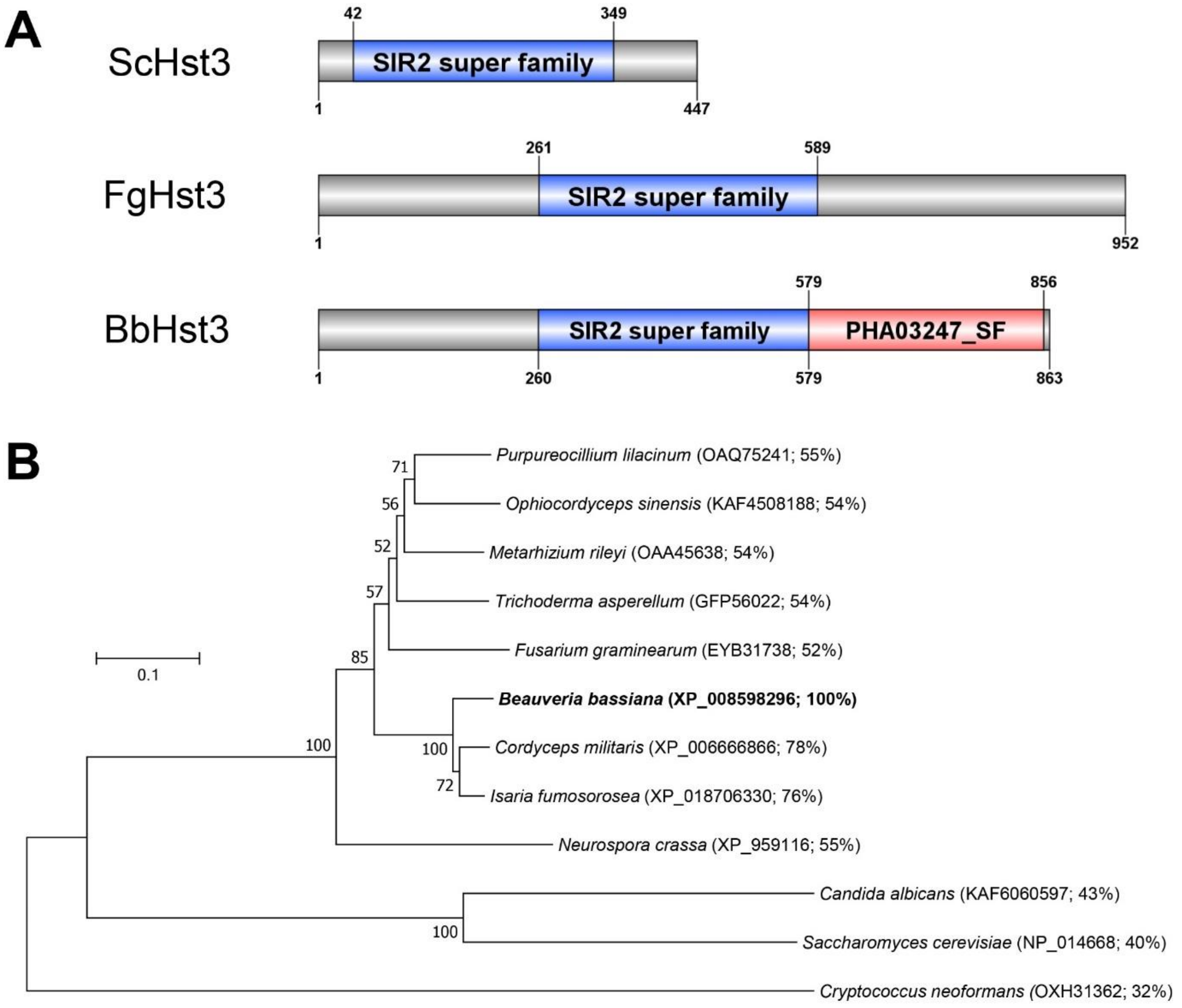

FIG S1. Bioinformatic analysis of fungal Hst3 homologues. (**A**) Sequence features of Hst3 homologs found in *Beauveria bassiana* (Bb), *Saccharomyces cerevisiae* (Sc), and *Fusarium graminearum* (Fg). The domain of each protein was predicted at https://blast.ncbi.nlm.nih.gov/Blast.cgi. (**B**) Phylogenetic relationship of *B. bassiana* Hst3 with the homologs found in other representative fungi. A neighbor-joining method in MEGA7 at http://www.megasoftware.net was used in the phylogenetic analysis. Each fungal name is followed by the NCBI accession code of each protein and its sequence identity (%) to *B. bassiana* Hst3 in parentheses. Poisson model was used with 1000 bootstrap replications in uniform rates. Scale bar: branch length proportional to genetic distance assessed with the neighbor-joining method.

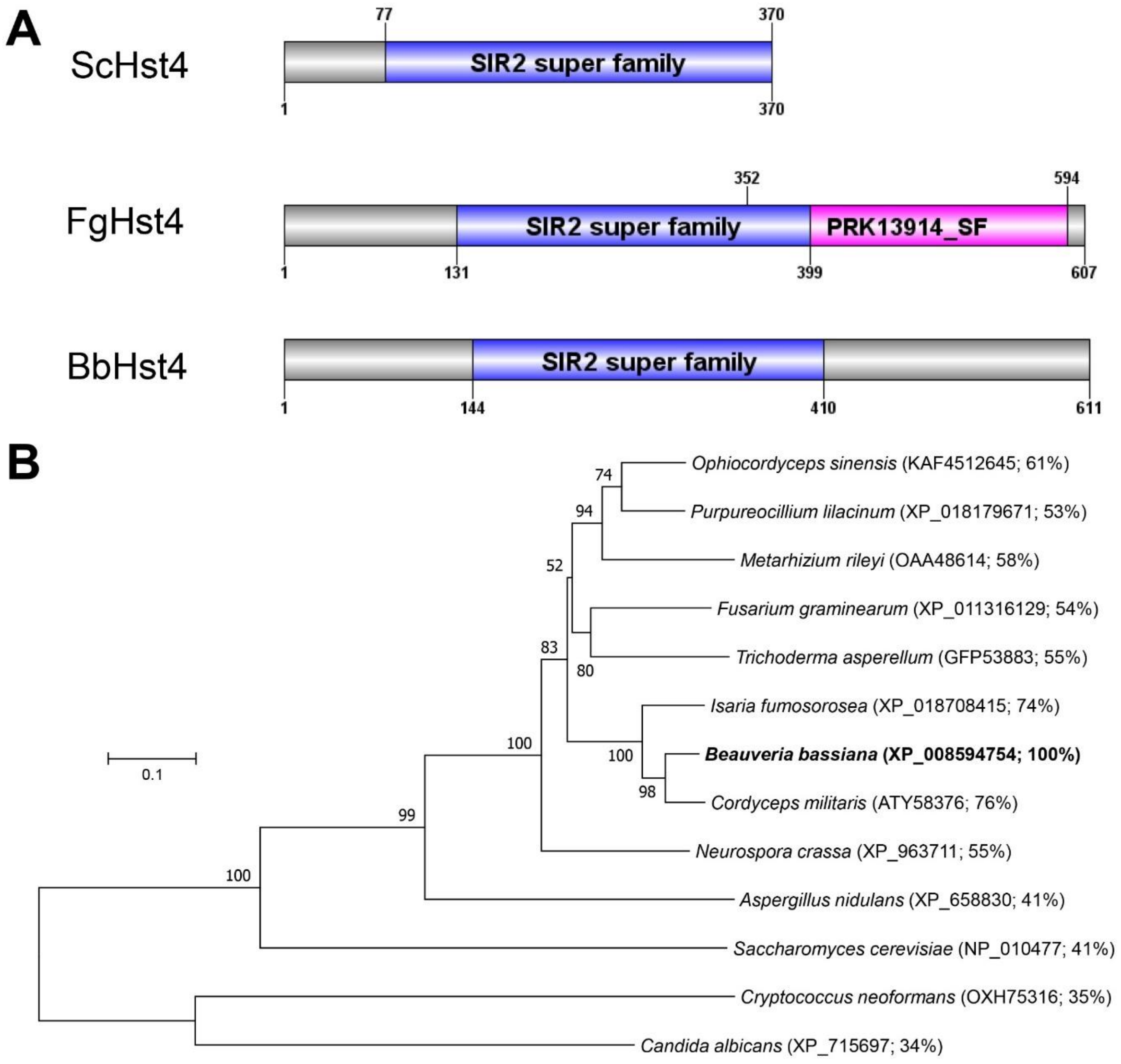

FIG S2. Bioinformatic analysis of fungal Hst4 homologues. (**A**) Sequence features of Hst3 homologs found in *Beauveria bassiana* (Bb), *Saccharomyces cerevisiae* (Sc), and *Fusarium graminearum* (Fg). The domain of each protein was predicted at https://blast.ncbi.nlm.nih.gov/Blast.cgi. (**B**) Phylogenetic relationship of *B. bassiana* Hst4 with the homologs found in other representative fungi. A neighbor-joining method in MEGA7 at http://www.megasoftware.net was used in the phylogenetic analysis. Each fungal name is followed by the NCBI accession code of each protein and its sequence identity (%) to *B. bassiana* Hst4 in parentheses. Poisson model was used with 1000 bootstrap replications in uniform rates. Scale bar: branch length proportional to genetic distance assessed with the neighbor-joining method.

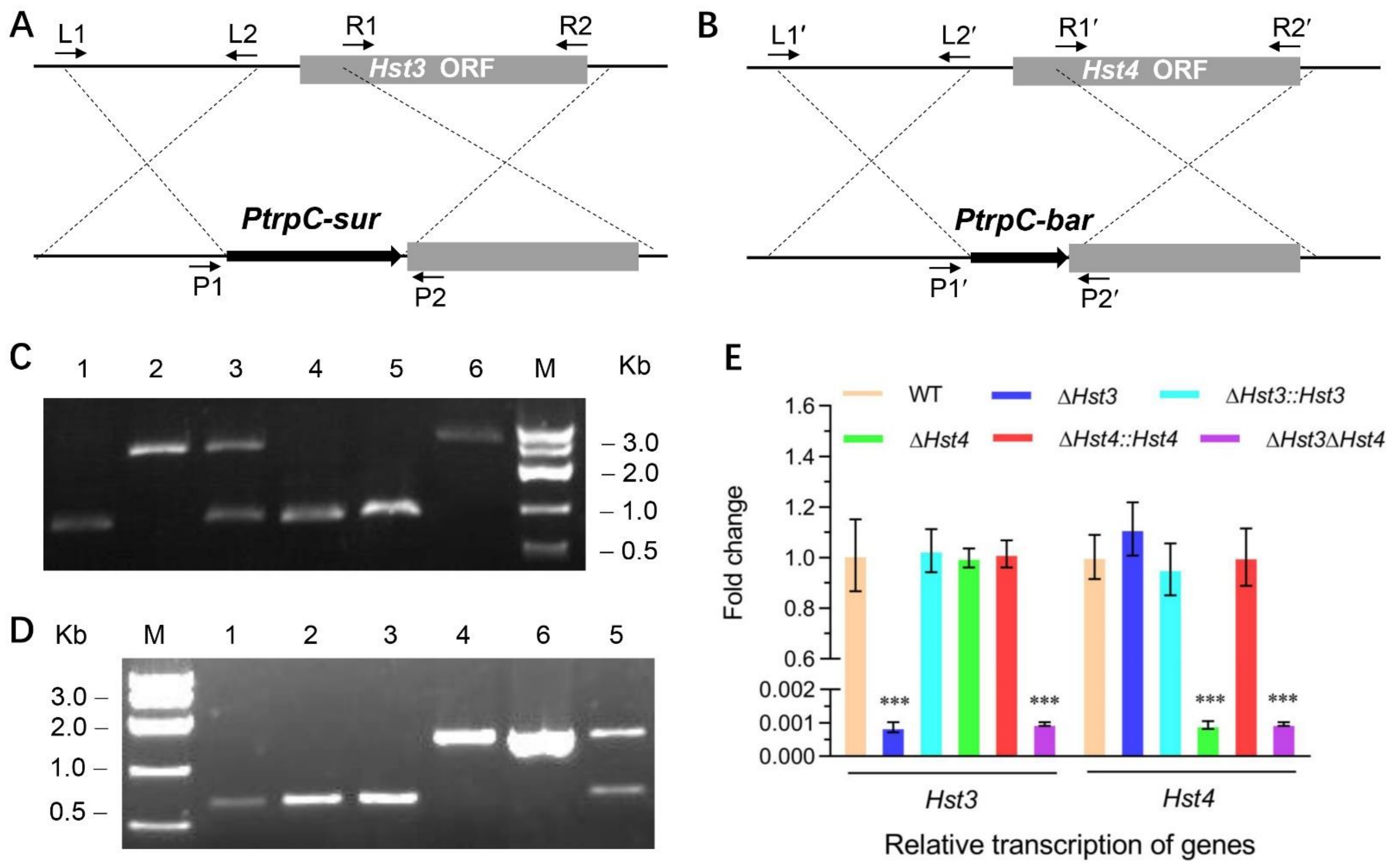

FIG S3 Construction and identification of *B. bassiana Hst3* and/or *Hst4* mutants. (**A, B**) Schematic diagram for the strategy of *Hst3* or *Hst4* deletion. (**C, D**) The verification of *Hst3* deletion/complementation (C) or *Hst4* deletion/complementation (D) via PCR with paired primers (Table S1). Lanes 1: wild-type; Lanes 2: Δ*Hst3* mutant; Lanes 3: Δ*Hst3*::*Hst3*; Lanes 4: Δ*Hst4* mutant; Lanes 5: Δ*Hst4::Hst4* mutant; Lanes 6: Δ*Hst3*Δ*Hst4* mutant. (**E**) The verification of *Hst3* or *Hst4* deletion/complementation via real-time PCR with paired primers (Table S1). Transcription analyses were performed three times. Error bars = ± SD. Asterisks indicate significantly different from unmarked (Tukey's HSD, *** indicates $p < 0.001$)

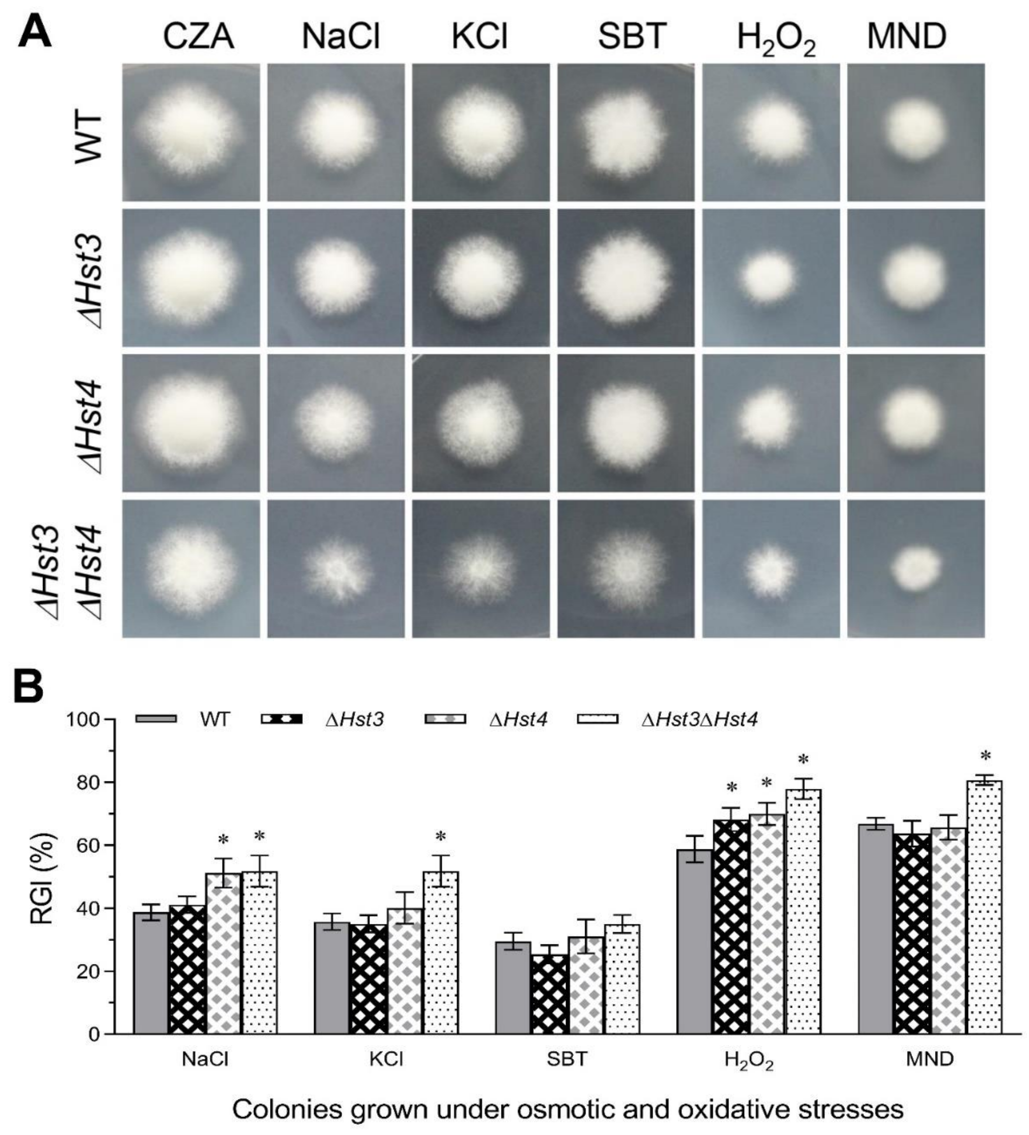

FIG S4 Impact of *BbHst3* and/or *BbHst4* deletion on fungal resistance to osmotic and oxidative stresses. (A,B) Representative images and quantification of relative growth inhibition (RGI) of fungal colonies grown at 25°C for 8 d on CZA supplemented with NaCl (0.4 M), KCl (0.4 M), sorbitol (0.8 M), H_2O_2 (2 mM), and menadione (MND; 0.02 mM). All colonies were initiated by spotting 1 µl of $1×10^6$ conidia/ml suspension on the plates. All experiments were performed three times. Error bars = ± SD. Asterisks indicate significantly different from unmarked (Tukey's HSD, * indicates $p < 0.05$).

TABLE S1 Primers used for genetic manipulation of *Hst3* or *Hst4* in *B. bassiana*.

Primers	Paired sequences (5'-3')*	Purpose
Hst3-up-F/R	AAAAACCCGGGAACAAGAAGATTGCTATGGCGA / AAAAAGGATCCAAGATTGAGTGTGTATGTCCGA	Cloning *Hst3* 5′-end (1535 bp)
Hst3-dn-F/R	AAAAACTCGAGCTGTTTTCTGCAGGATTTTCGA / AAAAAACTAGTAGTCAATAATGTCACCCCACGA	Cloning *Hst3* 3′-end (1583 bp)
Hst4-up-F/R	AAAAACCCGGGTATCTGTAGGTACGCTGGCATT / AAAAAGGATCCGTTGAGGTACTTGGTTGTGCG	Cloning *Hst4* 5′-end (1576 bp)
Hst4-dn-F/R	AAAAATCTAGATCTCCGACTCTACTCGCAAAAT / AAAAAGTTAACTCTCTGATGATTGGCTTTTGGA	Cloning *Hst4* 3′-end (1572 bp)
pHst3-F/R	CTTCAGTCACTTCAACGCCTAC / CTTCTTCCTCGTTCGTGCCAT	PCR detecting *Hst3*
Hst3-ORF-F/R	ggggACCACTTTGTACAAGAAAGCTGGGTNCCCAACTCCCAAGTCTGTCA / ggggACAAGTTTGTACAAAAAAGCAGGCTNCCATTCTTCTGCGGTGAGTG	Cloning *Hst3* full ORF
Hst3-ORF-F/R	ggggACCACTTTGTACAAGAAAGCTGGGTNAGAAGAGGTGAAGACCGAGAAG / ggggACAAGTTTGTACAAAAAAGCAGGCTNCCGTAGTAGGTAACGAGAGGAGT	Cloning *Hst4* full ORF
pHst4-F/R	CAGCCAATCTGACGACGGTAG / GAGAGGCTCCATTTGGGTATC	PCR detecting *Hst4*
18S-F/R	TGGTTTCTAGGACCGCCGTAA / CCTTGGCAAATGCTTTCGC	qPCR detecting 18S rRNA
pHst3-F/R	ACGACTTAGCACTCTATC / CTCTCACCATAACCTTGA	qPCR detecting *Hst3*
pHst4-F/R	AAGACGAGCAAGAATATG / TGGTAATGTCACAGTAGA	qPCR detecting *Hst4*

* Underlined regions denote the restriction enzyme sites used for deleting *Hst3* (*Xma*I/*BamH*I and *Xho*I/*Spe*I) or deleting *Hst4* (*Xma*I/*Hind*III and *Xba*I/*Hpa*I).
